# A new initiative for the development of new diagnostic tests for human African trypanosomiasis

**DOI:** 10.1186/1475-9292-5-1

**Published:** 2006-04-25

**Authors:** Dietmar Steverding

**Affiliations:** 1 Biomedical Research Centre, School of Medicine, Health Policy and Practice, University of East Anglia, Norwich NR4 7TJ, UK

## Abstract

Human African trypanosomiasis is a threat to millions of people living in sub-Saharan countries and is fatal unless treated. At present, the serological and parasitological tests used in the field for diagnosis of sleeping sickness have low specificity and sensitivity. There is clearly an urgent need for accurate tools for both diagnosis and staging of the disease. The Foundation for Innovative New Diagnostics and the World Health Organization have announced that they will collaborate to develop and evaluate new diagnostic tests for human African trypanosomiasis.

## Development of diagnostics for human African trypanosomiasis

### Background

Human African trypanosomiasis (HAT), also known as sleeping sickness, is caused by protozoan parasites of the genus *Trypanosoma*. Infection with *T. brucei gambiense *results in the chronic form of sleeping sickness in West and Central Africa, whereas *T. brucei rhodesiense *gives rise to the acute form of the disease in East and Southern Africa. If left untreated, the disease is fatal, and therefore early diagnosis is essential. However, existing diagnostic tests for HAT are not sensitive and specific enough, and are difficult to use in rural areas. Therefore, the Foundation for Innovative New Diagnostics (FIND) and the World Health Organization (WHO) held a joint meeting in Geneva, Switzerland, on February 6–7, 2006, to launch a new initiative for the development of new diagnostic tests to support the control of sleeping sickness. This initiative is funded by a grant of 10 million USD for the next 5 years from the Bill & Melinda Gates Foundation (BMGF). Representatives from FIND, WHO, BMGF, pharmaceutical companies, national control programs and the academic sector attended the meeting to discuss current problems and necessary improvements in the diagnosis of HAT.

Over 97% of all sleeping sickness cases reported in the past 5 years were *T. b. gambiense *infections (P. Simarro, personal communication). The common test for serodiagnosis of *T. b. gambiense *sleeping sickness is the Card Agglutination Test for Trypanosomiasis (CATT) [1]. However, this test does not detect *T. b. rhodesiense*. One problem with the CATT is its low specificity of 74–95% with a sensitivity of 87–98% [2-4]. Therefore, demonstration of the parasite in tissue fluids of a patient is strictly required before the initiation of chemotherapy, especially as some drugs used for treatment of sleeping sickness have serious side effects [5]. However, parasitological detection of trypanosomes in blood sample is often difficult because of relatively poor sensitivity of microscopic techniques and of low and fluctuating parasitaemias. Another problem in HAT diagnosis is the staging of the disease. The early stage of sleeping sickness is defined by the restriction of the parasites to the blood and lymph systems. The late stage of the disease is characterised by the presence of trypanosomes and an increase white blood cell (WBC) count in the cerebrospinal fluid (CSF). As there are no clear clinical criteria for early and late stage disease [6], staging relies on the examination of the CSF obtained by lumbar puncture which is painful, risky and complicated when performed under the field conditions. However, the number of trypanosomes in the CSF is usually scanty and WBC counts are variable. In addition, staging based on WBC counts is not standardised; the suggested cutoff ranging between 5 WBC/μl to 20 WBC/μl [7]. Hence, there is a need for new diagnostic tools that are simple and efficient in the detection of both stages of HAT.

### What are the technical options for the development of new and improved HAT diagnostics?

One possibility could be the development of an antibody detection test that uses an antigen different from that of the CATT (lyophilized *T. b. gambiense *bloodstream forms of the variable antigen type LiTat1.3). So far, there are about 20 potential antigens characterised, available as recombinant or synthetic polypeptides, which could be used for a serodiagnostic test for HAT. In addition, a different test format could be employed, e.g. lateral flow or gel agglutination. Finally, tests could be developed for the detection of both *T. b. gambiense *and *T. b. rhodesiense*. Development of an antigen detection test seems to be more difficult; while a test for the detection of circulating antigens in sleeping sickness patients has been described, the Card Indirect Agglutination Trypanosomiasis Test (CIATT) [8], there are some doubts about its specificity [9]. Nevertheless, other antigens, e.g. secreted enzymes, could be used in the development of an antigen detection assay. Another option for the development of new diagnostic tests for HAT could be the use of molecular methods. Although the implementation of PCR methods in the field is not feasible at the moment, a simplified PCR test may be possible for field use. The loop-mediated isothermal amplification (LAMP) reaction is a simple nucleic acid amplification (NAA) method that produces large amounts of target-specific DNA under isothermal conditions with a simple incubator [10]. Another advantage of the LAMP method is that DNA amplification can be monitored with the naked eye without the use of special detection devices. Given the extremely limited infrastructure in the settings in which HAT occurs however, there is still substantial progress to be made before NAA testing can be widely implemented as a diagnostic tool for HAT.

The development of tools for staging of HAT is much more difficult. One option could be a simplified CSF examination. For instance, tests could be designed that detect markers for inflammatory reactions of the brain (e.g. neurological autoantibodies, cytokines, intrathecal IgM [11,12]) or fragments of trypanosomes or nerve cells in the CSF, although the latter occurs very late in the infection. The development of a staging test that does not require CSF sampling is even harder. One possibility could be the detection of neurological antigens in the blood. However, this requires research into the identification and characterisation of such antigens in the first place. Another interesting non-invasive staging method is polysomnography, the monitoring and recording of physiological data during sleep. It has been shown that late stage sleeping sickness patients have a disturbed circadian distribution of the sleep-wake episodes as well as an altered sleep structure with frequent sleep onset rapid eye movement periods (SOREMPs) [13]. Importantly, early evidence suggests that SOREMPs appear soon after the central nervous system invasion by trypanosomes. However, polysomnography is a demanding technique which has to be shown that it can be used routinely under field conditions.

### What are the roles of FIND and WHO in the development of new diagnostic tools for HAT?

FIND is an independent and a non-profit foundation based in Geneva [14]. FIND's activities in this initiative will be focus on the development, evaluation and demonstration of new HAT diagnostics. First, FIND will work in partnership with academic and industry groups to develop promising diagnostic assays into applicable products. Next, FIND will establish a formal mechanism to evaluate these products in field trials. Finally, FIND will collaborate with WHO, other agencies and national programmes to demonstrate the impact that use of these products has on HAT control. In other words, FIND will create a network of public and private partners to produce new diagnostic tests for HAT and to demonstrate their effectiveness. For upstream research discovery and downstream implementation activities FIND will liaise with the relevant partners.

The role of WHO in this initiative is to establish a network of experienced diagnostic centres in Africa with the capacity to enrol 300 – 500 confirmed sleeping sickness patients per year and to characterise samples from up to 1000 endemic controls. In addition, the WHO will ensure that the diagnostic capacity is maintained in study areas and that national HAT control programmes are fully integrated into the evaluation of the new tests. Furthermore, the WHO will establish a HAT specimen bank by collecting reference materials from well characterized patients and controls, assigning a professional facility to be responsible for cryopreservation, and managing the distribution of specimens to support the development and validation of new tests. Finally, the WHO will provide training in the application and evaluation of the new diagnostics and give operational support to the conduct of field trials.

## Conclusion

FIND and WHO have started a collaboration to develop urgently needed new diagnostic tools for HAT. The new tests should ideally be simple (easy to be used by minimal trained staff in the field), rapid (to be carried out in less than 30 min), efficient (better than the current diagnostics) and cheap (less than 1 USD per person). It is hoped that the new tests will allow for early case detection and simplified staging that will improve disease management and ultimately support the elimination of sleeping sickness as a public health problem.

## Competing interests

The author declares that he has no competing interests.
